# Effects of the Combinations of Rhizobacteria, Mycorrhizae, and Seaweed, and Supplementary Irrigation on Growth and Yield in Wheat Cultivars

**DOI:** 10.3390/plants10040811

**Published:** 2021-04-20

**Authors:** Z. Najafi Vafa, Y. Sohrabi, R. Z. Sayyed, Ni Luh Suriani, Rahul Datta

**Affiliations:** 1 Department of Agronomy and Plant Breeding, Faculty of Agriculture, University of Kurdistan, Sanandaj 66177, Iran; y.shorabi@uok.ac.ir; 2 Department of Microbiology, PSGVP Mandal’s Arts, Science and Commerce College, Shahada 4245409, Maharashtra, India; 3 Biology Study Program, Mathematics and Natural Sciences Faculty, Udayana University, Bali 80361, Indonesia; niluhsuriani@unud.ac.id; 4 Department of Geology and Pedology, Mendel University in Brno, Zemedelska 1, 613 00 Brno, Czech Republic

**Keywords:** biofertilizers, drought stress, mycorrhiza, irrigation, wheat

## Abstract

Wheat is a staple food consumed by the majority of people in the world and its production needs to be doubled to feed the growing population. On the other hand, global wheat productivity is greatly affected due to drought and low fertility of soil under arid and semi-arid regions. Application of supplementary irrigation and plant growth-promoting rhizobacteria (PGPR) has been suggested as sustainable measures to combat drought stress and to improve soil fertility and, hence, crop yield. This research was undertaken to study the effect of supplementary irrigation together with a combination of various PGPR on the growth and yield of two wheat cultivars, namely Sardari and Sirvan. The results of variance analysis (mean of squares) showed that the effect of irrigation, cultivar, and irrigation and biofertilizer and irrigation on height, spike length, seed/spike, and numbers of spikes/m^2^, 1000-seed weight, and grain yield were significant at 1% probability level. The effect of cultivar and irrigation interactions showed that the highest grain yield was obtained in a treatment with two additional irrigations in Sirvan cultivar (5015.0 kg/ha) and Sardari (4838.9 kg/ha) as compared to the 3598 kg/ha and 3598.3 kg/h grain yield in Sirvan and Sardari cultivars with similar treatment, but without irrigation, i.e., dryland farming. Drought conditions significantly affected the wheat grain yield while supplementary irrigation resulted in 39.38% and 34.48% higher yields in Sirvan and Sardari cultivars.

## 1. Introduction

Bread wheat (*Triticum aestivum* L.), is the most important annual crop and has the largest area of dryland cultivation in the world [1,2,3,4]. Approximately 90% of the wheat produced in the world is bread wheat and 10% is durum wheat. Wheat has extraordinary significance in human nutrition as it is considered an important measure to combat hunger [5], and it provides about 20% of the calories, proteins, minerals, and B vitamins [2]. However, in dryland cultivation, the productivity and yield of wheat are severally affected by drought and low soil fertility [6]. Moreover, global climate changes are increasing the incidence of drought stress [7]. These situations demand sustainable management strategies [8]. Application of plant growth promoting rhizobacteria (PGPR), mycorrhizae, and seaweed, along with supplementary irrigation, have been suggested as sustainable strategies to combat the drought stress and low fertility of soil [9,10,11].

Application of PGPR offers numerous advantages, in terms of plant growth promotion through the secretion of various plant beneficial metabolites [12], increase in soil fertility [13], improved drought tolerance in plants through the production of exopolysaccharides and osmolytes [1,14,15], protection of plants from pathogens through the production of antibiotics, hydrogen cyanide, hydrolytic enzymes, etc. [16,17,18]. Moreover, it induces resistance in plants by triggering various jasmonic acid and other pathways [19,20,21,22,23].

The symbiosis of arbuscular mycorrhizal (AM) fungi with plant roots helps plants tolerate and overcome drought stress [24], AM improves physiological status plants under drought stress through better uptake of soil nutrients and water, reduced oxidative damage, enhanced root water transport capacity, or facilitated switching between apoplastic, and cell-to-cell water transport pathways [25]. AM symbiosis has proved useful in various crops, including maize and wheat [25].

The addition of seaweed functions as a biostimulant to improve plant growth and as an amendment to boost crop yield, as it contains a number of plant growth-stimulating compounds, such as macronutrient and microelement nutrients, growth hormones, amino acids, vitamins, betaines, cytokinins, and sterols [26]. Seaweed extracts induce changes in the physiological and biochemical process, leading to improved nutrient uptake and better plant growth [26]. They induce early seed germination, improve root growth, increase leaf chlorophyll, improve crop yield, and enhance resistance to biotic and abiotic stress [26]. In addition, seaweed extracts also improve the physico-chemical, and biological properties of soil [26]. The addition of seaweed extract has proved useful in tomato, maize, wheat, etc. [27].

Therefore, consortia of PGPR, AM, and seaweed extract, along with supplementary irrigation, offers multiple advantages [24]. Since the effects of co-inoculation of consortia of PGPR + seaweed extract with supplementary irrigation on wheat in rainfed and irrigated conditions have not been investigated so far, the present research was undertaken to study the effect of supplementary irrigation, combinations of PGPR, and seaweeds, and types of cultivars on growth and yield improvements in wheat.

## 2. Materials and Methods

### 2.1. Microbial Cultures and Wheat Varieties 

Phosphozist that contained phosphate solubilizing *Phosphobacteria* sp., (6×10^8^ cells/mL) and nitrozist that contained nitrogen-fixing *Azospirillum* sp. and *Azotobacter* sp. (6 × 10^8^ cells/mL) were procured from Green Biotechnology, Iran. Mycopresica that contained AM fungus, *Glomus mosseae* (5 × 10^6^ spores/mL of) was obtained from Turan Shahroud Biotechnology Co., Iran, under the brand name. Two wheat cultivars, namely Sardari and Sirvan Sanandaj, were obtained from the local market in Sanandaj, Iran.

### 2.2. Analysis of Physicochemical Properties of Soil 

A composite sample of farm soil was prepared from soil randomly collected from depths (0–30 cm) followed by the measurement of soil texture [28], and the electrical conductivity [29], the potassium content [30], phosphorus content [31], organic carbon, and the total nitrogen contents [32].

### 2.3. Treatments

The present research was conducted on a wheat field in the research farm of the Faculty of Agriculture of Kurdistan University, Dehgolan, Iran (47.18° East and 35.18° N). Two wheat cultivars were sown in a split-plot (twice split plots) in a randomized complete block design (RBD) with five replications in two cropping years (2017–2018 and 2018–2019). The main factor was supplementary irrigation at three levels

- Control—dryland farming;
- Irrigation at the shoot stage;
- Irrigation at the spike stage.

Various combinations of the following treatments were applied to the seeds:

Control (no biofertilizers);
Mycorrhiza;
Seaweed extract;
Nitrozist and phosphozist;
Mycorrhiza + nitrozist and phosphozist;
Seaweed extract + nitrozist and phosphozist;
Mycorrhiza + seaweed extract;
Mycorrhiza + phosphozist + nitrozist + Seaweed extract.

### 2.4. Plots

The dimensions of each sub-plot was 6 × 1.20 m^2^. Each experimental plot had six planting lines of six m, and a row spacing of 20 cm. Planting density was kept at 450 seeds/m^2^ and seeds were planted at a depth of 3–5 cm. The distance between replicates and main plots was 2 m, which was randomly placed in the subplots. The distance of each subplot from the other subplot was considered as three planting lines. The distance between each subplot was considered to be 20 cm (one planting line).

### 2.5. Application of PGPR, AM and Seaweeds

A total of 60 kg of seeds of each cultivar were soaked in one L each of P solubilizing (phosphozist) and nitrogen-fixing (nitrozist) bacterial preparation for 8 h, dried in shades, and used for sowing [33]. Mycorrhizae fungus, *G. mosseae,* was applied at the rate of 40 g/m^2^ as a strip next to the seeds at planting [34]. Seaweed extract was applied as a foliar spray at the rate of 3 L/ha during three growth stages, i.e., vegetative shoot growth, spike growth, and pollination [35].

### 2.6. Irrigation of Experimental Plots

Irrigation of experimental plots at each stage was calculated after removing the roots from several plots. These root preparations were dried in an oven at 70 °C for 94 h and the moisture content was calculated as follows [36]:

(1) $$\mathrm{Moisture}\;\left(\%\right)=\frac{\mathrm{Intial}\;\mathrm{soil}\;\mathrm{weight}-\mathrm{Soil}\;\mathrm{dry}\;\mathrm{weight}}{\begin{array}{c} \;Soil\;dry\;weight\; \end{array}}$$

The irrigation volume (V) for each stage of supplementary irrigation treatments was calculated from the following formula [36].

(2) $$\mathrm{Irrigation}\;\mathrm{volume}\;=\frac{\left(\mathrm{Fc}-m\right)\;\times \;\mathrm{pa}\;\times \;a\;\times \;\mathrm{ds}}{E}$$

where:

V is the volume of irrigation in cubic meters;
Fc is the percentage of soil moisture in the field capacity;
M is the percentage of soil moisture before irrigation, a is the specific gravity of the soil;
A is the area of the main plot;
ds is the depth of root development in centimeters at the desired stage; and
Ea is the irrigation efficiency, which in this experiment is considered 45%.

### 2.7. Measurement of Morphological Traits

The morphological traits, such as plant height, tiller number, awl length, peduncle length, spike length, spike weight, number of seeds per spike, number of spikes per square meter, 1000-seed weight, and grain yield were measured after harvesting 1 m^2^ from each plot scrubbing (by removing the fringe effect). The plants and their foliage were measured by using a scale with an accuracy of 0.01. The plant components, such as leaves, stems, and spikes were measured as fresh weight. The length of the main spikes up to the tallest awn was measured as the length of the spike. To measure the number of seeds per spike, 10 spikes from each plot were randomly selected, threshed (manual threshing), and the number was divided by the total number of spikes. Grain yield was calculated by weighing the total seeds obtained from an area of one square meter in each experimental unit. The number of spikes per square meter was measured with a box in one square meter of each plot, and the number of spikes in the box was counted as spikes per square meter. Weight of spikes was measured from 10 randomly selected spikes using a scale [37].

### 2.8. Statistical Analysis

All experiments were performed in five replicates and the average of five replicates were compared by the LSD test and one analysis of variance (ANOVA). All statistical operations were performed with the help of SAS statistical software version 9.1.

## 3. Results

### 3.1. Physicochemical Properties of Soil

The analysis of physicochemical parameters of soil revealed a significant improvement. A significant improvement in the electrical conductivity, pH, available P, K, total organic carbon, and total nitrogen content of farm soil was recorded during the second year of cropping (Table S1).

### 3.2. Plant Growth and Yield Parameters

#### 3.2.1. Plant Height

The results of the combined analysis showed that the effect of irrigation, the effect of irrigation x cultivar interaction, the effect of biofertilizer and irrigation interaction, and the effect of year × cultivar interaction were significant at the level of one and five percent, respectively (Table 1). The results of comparing the mean of interactions of a cultivar with year showed that the first cropping year was higher than Sardari and Sirvan cultivars compared to the second cropping year. The results of the comparison of the mean of interactions between cultivar × irrigation showed that the highest plant height was observed under double irrigation in Sardari and Sirvan cultivars, which were 7.2 and 8.6% higher than the value of this trait in dryland farming conditions, respectively. In both wheat cultivars, the plant height increased with increasing irrigation frequency (Table 2). Comparison of two cultivars also showed that the height of Sardari wheat cultivar was higher than Sirvan wheat cultivar (Table 3). Sardari wheat cultivar, in comparison with Sirvan wheat cultivar, showed more water absorption power in rainfed conditions (Table 3). Biofertilizer application with irrigation (I × B) resulted in maximum plant height (67.44 cm).

#### 3.2.2. Number of Tillers

The results of the combined analysis of tiller numbers showed that only the effect of biofertilizers was significant at the level of one percent (Table 1). This treatment yielded maximum number of tillers, i.e., 6.15.

#### 3.2.3. Awn Length

The effect of irrigation, year, cultivar, irrigation interaction, and cultivar was significant at the level of one percent, and the effect on irrigation interaction and cultivar was significant at the level of five percent (Table 1). Comparison of the two-year mean values of the effect of cultivar and irrigation interaction on abrasion length shows that the amount of abrasion length in the second crop year in two irrigations in Sirvan cultivar was higher as compared to the first crop year (Table 4). The study of the effect of year on awning length in wheat cultivars shows that the length of an awn in the second crop year was higher compared to the first crop year. The awn length during second year was 5.24 cm and 4.55 cm vis-a-vis 4.96 cm and 4.55 cm during first year in for Sardari cultivar and Sirvan cultivar, respectively (Table 3).

#### 3.2.4. Length of Peduncle

The effect of irrigation, the effect of irrigation × year, and the effect of irrigation and cultivar on peduncle length were significant at the level of one percent (Table 1). Comparison of the two-year average data shows that no irrigation (drought stress/dryland farming) significantly reduced peduncle length. The maximum peduncle length was evident in the second year and twice irrigation (40.36 cm), which was 9% more than the peduncle length in the first year and dryland conditions (37.04 cm) (Table 2). The effect of cultivar and irrigation interaction resulted in the highest peduncle length in Sardari (43.44 cm) as compared to Sirvan cultivar (40.20 cm) with two supplementary irrigations, i.e., 17.7 and 9.3% higher than the peduncle length in Sardari (36.91) and Sirvan (36.78) cultivars under dryland conditions (Table 4).

#### 3.2.5. Length of Spike

The effect of irrigation and the effect on irrigation and cultivar interactions were significant at the level of one percent, while the effect of biofertilizers on the interaction of biofertilizers and irrigation and the effect on cultivar and biofertilizer interaction were significant at level five percentage (Table 1). Comparison of the mean effect on cultivar and irrigation interaction shows the highest spike length obtained in treatment with two irrigations and Sardari and Sirvan cultivars with average (16.85 cm) and (18.20 cm), respectively, which was 28.3 and 30% more, as compared to the treatment without irrigation of rainfed cultivation (Table 4). The mycorrhizae + seaweed extract + nitrozist and phosphozist and interaction of cultivar resulted in the highest spike length in the Sardari wheat, with averages of 15.90 cm, and the Sirvan wheat length, 16.38 cm. A 6.14% and 12.88% more spike length was obtained in biofertilizers (control) treatments in Sardari (14.98 cm) and Sirvan (14.51 cm) cultivars, respectively (Table 5).

#### 3.2.6. Weight of the Spike

The spike weight data showed that the effect of irrigation and the interaction effect of biofertilizers and cultivar and irrigation were significant at one percent level while the effect of cultivars and biofertilizers was significant at five percent level (Table 1). Three-way interaction of irrigation and cultivar and biofertilizers showed that the double irrigation and application of biofertilizers+ mycorrhizae + seaweed extract resulted in the 48.0% and 42.6% higher weight in both the cultivars as compared to the control treatment (without biofertilizer) (Table 5).

#### 3.2.7. Number of Seeds per Spike

The effect of irrigation, biofertilizers, the effect of irrigation and cultivar interaction, the effect of year and cultivar interaction and the effect of biofertilizer and irrigation interaction were significant at one percent level and the effects of year and interaction of biofertilizers and cultivar and irrigation were significant at the 5% level (Table 1). The highest number of seeds per spike were obtained with two additional irrigations in Sirvan and Sardari cultivars (Table 4). The effect of year cultivar on the number of seeds per spike in both wheat cultivars shows that the number of seeds per spike in the first crop year was higher as compared to the second crop year. The highest and lowest rates were observed for Sirvan cultivar in the first crop year and Sardari cultivar in the second crop year, respectively, with an average of 12.28 and 34.26 (Table 3). The effect of tripartite interaction of irrigation and cultivar and biofertilizers shows that the two treatments of supplementary irrigation and mycorrhizal fungus + seaweed extract + biofertilizers resulted in a 63.14% and 97.3% more seeds per spike in Sirvan and Sardari cultivars, respectively, over the control treatment (Table 5). The number of seeds per spike in a wheat plant in both cultivars (Sardari and Sirvan) in the system of two supplementary irrigations with treatments of mycorrhizal fungus + seaweed extract + nitrozist and phosphozist was greatly improved as compared to control under stress (dry land farming) without biofertilizers.

#### 3.2.8. Number of Spikes per Square Meter

The effect of irrigation, cultivar, the effect of interaction between irrigation and cultivar, and the effect of interaction between biofertilizers and irrigation were significant at the one percent level (Table 1).

The highest number of spikes per square meter was obtained in the interaction between cultivar and irrigation under double irrigation in Sirvan cultivar (641.33). It resulted in 23.6% more spikes obtained in the control treatment (dry land farming) (Table 4). In general, the results of this study showed that the application of biofertilizers (mycorrhizal fungus + algae extract + nitrozist and phosphozist) with two additional irrigations in both cultivars, respectively, increased the number of spikes per square meter by 27.0% and 13.4% as compared to one supplementary irrigation respectively. Moreover, applying one supplementary irrigation in the spike stage increased the number of spikes per square meter in Sardari and Sirvan cultivars by 20.9% and 10.3%, respectively, compared to the treatment without irrigation (dry land farming) (Table 5).

#### 3.2.9. 1000-Grain Weight

The results of combined analysis of 1000-grain weight data showed that the effect of year, irrigation, cultivar, the effect of interaction between irrigation and cultivar, the effect of the interaction of biofertilizers and irrigation were significant at one % level while the effect of the interaction of biofertilizers and cultivar and irrigation was significant at five % level (Table 2).

The effect of interactions between cultivar and irrigation on 1000-seed weight in one irrigation treatment and two irrigation treatments were 41.94 g and 43.34 g, respectively, which was 5.13 and 8.65 higher than the 1000-seed weight obtained in rainfed cultivation treatment (39.89 g) (Table 4). The three-way interaction of irrigation and cultivar and biofertilizers showed that the amount of 1000-seed weight in both Sirvan and Sardari cultivars in double irrigation and application of mycorrhizal fungi + seaweed extract + nitrozist and phosphozist had the highest 1000-seed weight. The lowest 1000-seed weight in the control treatment (no use of fertilizer sources) was observed in both Sardari cultivar (38.38 g) and Sirvan cultivar (37.75 g), which were 14 and 14.69, respectively. The percentage less than the amount the measured 1000-seed weight in the two irrigation treatments and the application of biofertilizers of mycorrhizal fungus + seaweed extract + nitrozist and phosphozist were in Sardari (44.63 g) and Sirvan (44.25 g) cultivars (Table 5).

#### 3.2.10. Grain Yield

The results of the combined analysis showed that the effect of irrigation, fertilizer, the effect on irrigation interaction and year, the effect of irrigation interaction and cultivar, and effect on the interaction of biofertilizers and irrigation on grain yield were significant at one percent level (Table 1). Comparison of the mean of two years shows that drought stress (dryland farming) significantly reduced grain yield. The highest grain yield was observed in the first year and twice irrigation (50,003.75 kg/ha) and the lowest grain yield in the second year and dryland farming conditions (3508.59 kg/ha) (Table 2). The effect of cultivar and irrigation interaction shows that the highest grain yield related to the treatment with two additional irrigations in Sirvan cultivar (5015.0 kg/ha) and Sardari (4838.9 kg/ha) was observed. This was 38.39% and 34.48% higher than the grain yield in the treatment obtained in Sirvan cultivar (3598 kg/ha) and Sardari cultivar (3598.3 kg/ha) without irrigation of dryland farming (Table 4).

## 4. Correlation Coefficient

Traits, such as the number of spikes per square meter, had a positive and significant correlation with grain yield; traits, such as spike length and spike weight, had a significant and negative correlation with grain yield. There was a significant and positive relationship between the number of tillers and the number of spikes per square meter, so that with increasing the number of tillers, the number of spikes per square meter increased. Moreover, the correlation coefficient showed that there was a positive and significant relationship between the number of spikes per square meter and grain yield at the level of 1%, which increased with increasing the number of spikes per square meter (Table S2). The relationship between spike weight and the number of spikes per square meter was a significant and negative effect that became significant at the level of one percent. This relationship may be due to the increase in the number of spikes per unit level of competition between plants for water absorption. Moreover, food increased, and as a result, the weight of the spike decreased (Table 2).

## 5. Discussion

Fallah [38] reported that with increasing drought stress intensity, plant height decreases. Jafari et al. [39] reported the highest (73.83 cm) height of the wheat plant in supplementary irrigation conditions during the flowering stage and the lowest (64.66 cm) in non-irrigation conditions. Alipanah et al. [40] observed a decrease in the height of wheat from 78 cm under full irrigation to 71 cm under drought stress. Mycorrhiza has a positive effect on wheat plant height, it increases photosynthesis by increasing water and nutrient uptake, and this leads to more asymmetry and improved plant growth/height as compared to un-inoculated plants [41]. Application of biofertilizers, especially *Azotobacter*, increase root development, and better absorption of water and nutrients, followed by vegetative growth and plant height [42,43]. PGPR also improves plant root development and thus increases water and nutrient uptake; they produce phytohormones, improve nitrogen status, and result in increased vegetative growth and plant height [44]. Seaweed extract also improves plant height [45] vegetative growth, leaf number, plant dry weight, and plant height [46]. It seems that the longer growth period of the Sardari cultivar has increased the distance between nodes, which ultimately leads to higher plant height in this cultivar.

Drought stress has been reported to cause a significant reduction in the number of wheat tillers [47]. The combined application of P solubilizing and nitrogen fixing PGPR has been reported to increases the number of tillers. Tahir et al. [48] reported that the application of *Azotobacter* sp. increases the number of tillers, the development of the root system, and increases the number of fertile tillers in wheat. Amrayi et al. [41] showed that the dual interactions of *Azotobacter* sp. and mycorrhiza in the cultivar and the triple interaction of *Azotobacter* sp. in mycorrhiza in cultivar at the level of one percent were significant on the number of fertile tillers. There was a significant difference between the mean dual interactions between mycorrhiza and *Azotobacter* sp. inoculation treatment (2.19 fertile tillers) as compared to least number of tillers in control treatment (1.83 fertile tillers) [41]. Golestani et al. [49] reported that the foliar application of seaweed extract on barley increases the number of tillers per plant, number of fertile tillers per plant, cluster length, and 1000-seed weight. The application of seaweed extract stimulate higher germination, root system growth as well as increased leaf area, leaf number, tillering, and plant weight. These effects increase plant height, the number of tillers, and the weight of rice stalks [50,51,52,53,54,55]. Seaweed extract is widely used as an enhancer to stress tolerance [56,57,58,59].

The higher length of an awn in the second crop year is due to changes in weather conditions during the two year [60,61,62,63]. This difference is due to the genetic characteristics of the Sardari cultivar compared to the Sirvan cultivar. Sardari cultivar is a dry and drought-tolerant cultivar whereas Sirvan cultivar is sensitive to moisture stress. Jiriai et al. [64] reported that inoculation of seeds with nitrogen-fixing PGPR improves flag leaf length and apricot length by 19% and 21%, respectively.

Mahato et al. [64] showed that inoculation of the wheat plant with *Azotobacter* sp. increased panicle length and peduncle length as compared to the control. This increase in panicle length and peduncle length may be due to beneficial effects of PGPR such as an increase in soil organic matter content, increase in soil NPK level, increase in soil fertility, and increased availability of nutrients for plant uptake, which in turn improves wheat growth.

Jiriaei et al. [65] and Safari [66] reported that the length of the wheat spike decreases with the increasing intensity of drought stress. Miraz et al. [67] showed that the interactions of the two factors of biofertilizer application and phosphorus fertilizer also had a significant difference. The length of the spike in the seeds treated with PGPR: water (1:1) has the highest length as compared to the length of the spike in the seeds treated with 100% inorganic fertilizers. Miraz et al. [66] showed that inoculation of seeds with PGPR increases the absorption of nutrients, especially phosphorus that caused an increase in the length of the spike. Ghaffarizadeh et al. [68] reported that the use of algae extracts increases the length of the wheat spike as compared to the control treatment. Larger spikes may play a role in increasing yield due to more photosynthesis in the non-grain structures of the spike. Regarding the distribution of photosynthetic materials made between the spikes, it is observed that as the physiological maturity stage approaches, the percentage of the total weight of the biomass formed by the spikes changes, and the weight of the spikes (without seeds) gradually decreases [69].

The number of seeds per spike in a wheat plant is one of the traits that are affected by drought stress in the reproductive stage even before the plant enters the pollination stage. There is a negative correlation between the number of grains and the weight of 1000 grains of wheat, which indicates a decrease in the share of carbohydrate transfer to each grain as a result of increasing the number of grains [70]. Rahimi et al. [71] reported that under drought stress conditions, the number of seeds per spike, 1000-seed weight, and spike weight, have the most direct effect on grain yield in drought stress conditions. Amrayi et al. [41] found significant improvement at a 1% probability in spike weight due to interaction of *Azotobacter* sp. and *Azospirillum* sp. and mycorrhiza. The increase in grain weight in the wheat plant due to bacterial inoculation is attributed to the positive role of PGPR on root growth, which helps in more absorption of water and nutrients [72].

Jafari et al. [65] reported the highest number of spikes (539) with supplementary irrigation at the flowering stage as compared to the lowest number (448) of spikes obtained under dryland farming conditions. Sayyahfar et al. [73] reported the highest number of spikes per square meter (573.3) in inoculation treatment with mycorrhizal fungus as compared to the lowest number of spikes per square meter (4.4 563) obtained in the control treatment. Cabral et al. [74] reported an increase in the number of spikes per plant following the applications of mycorrhizal fungus. Tavakoli and Jalali [75] reported a positive effect of combined application of nitrogen fertilizer on the number of wheat spikes per square meter as a result of improved root development that caused more uptakes of water and nutrient. Salim et al. [76] reported that the use of algae extracts (2 g/L) increased the number of tillers, length, length of the main spike, number of seeds per spike, and wheat spike compared to control plants (no application of algae extract).

Vahamidis et al. [77] also reported that compared to the control (full irrigation), depending on the year, genotype, and water availability, and the number of seeds per spike among durum wheat cultivars decreased from 4.4 to 12.7%.

The effect of drought stress on 1000-seed weight varies with cultivars and with drought intensity. Jafari et al. [39] reported that supplementary irrigation at the flowering stage with 51.16 g/m^2^ and dryland treatment without supplementary irrigation at 44.05 g/m^2^ had the highest and lowest 1000-seed weight, respectively. Weight loss of 1000 seeds in dryland treatment can be attributed to the lack of sufficient moisture that prevents the absorption of nutrients and causes decreased photosynthesis. This reduction in turn affects the grain food reserves and 1000-grain weight [39]. Jokar et al. [63] found that 1000-seed weight was reduced in rainfed conditions compared to supplementary irrigation conditions.

Rezaei et al. [78] reported that grain yield is affected by the type of fertilizer used. Hou et al. [79] found that yield losses under drought stress are mainly due to a decrease in the number of grains per spike in wheat. The superiority of the combined fertilizer treatment can be attributed to better nutrition and a gradual supply of nutrients required by the plant and consequently increased photosynthesis and plant material production [39]. Azarmehr et al. [80] reported an 11% increase in 1000-seed weight of rapeseed under the influence of seaweed extract.

Ahmadi et al. [81] reported decrease in the mean traits of plant height, the number of plants per square meter, cluster length, peduncle length, filling period length, grain yield, and 1000-seed weight under non-irrigated conditions as compared to irrigation conditions. Ahmadi et al. [81] showed that water stress in durum wheat reduced yield by 24.04% on average. Supplementary irrigation in the flag pod swelling stage (Booting) with the application of biofertilizers had the highest wheat grain yield in rainfed conditions [82]. The study of the effect of AMF fungi on wheat showed a 16% increase in yield in plants inoculated with AMF fungi [83]. Inoculation of AMF in winter wheat plants increases grain yield [84]. The plant roots and the microbial community influence each other [85]. Soil rhizobacteria plays an significant in nutrient uptake and plant growth [86,87,88,89,90]. Hamidi and Marashi [91] showed that increasing the use of phosphorus fertilizer and the use of mycorrhizal fungi can be effective in achieving the desired yield of wheat. *Azotobacter* sp. and *Azospirillum* sp. inoculation increased the percentage of grain yield as compared to the control. The positive correlation coefficient between the number of tillers and the number of spikes per square meter and a significant negative correlation between spike weight and the number of spikes per square meter may be due to the increase in the number of spikes per unit level of competition between plants for water absorption and food.

Decrease in grain yield is due to drought conditions that affects the absorption of water and minerals and hence affects the photosynthesis. Decreased photosynthesis resulted in a decrease in the number of grains. Thus, water deficit conditions affect the number of grains in wheat. Hou et al. [79] found that yield losses under drought stress are mainly due to a decrease in the number of grains per spike in wheat.

## 6. Conclusions

Application of combination of *Phosphobacteria* sp. + *Azotobacter* sp. and *Azospirillum* sp., mycorrhizal fungus and seaweed extract improves growth parameters and grain yield in wheat. A combination of these three in conjunction with supplementary irrigation offers a sustainable approach in managing drought stress while improving growth parameters and yield in wheat in semi-arid regions of the Kurdistan province of Iran.

## Figures and Tables

**Table 1:** Analysis of variance (mean of squares) of the physiological traits of wheat under the effect of supplementary irrigation and biofertilizers in the studied traits.

S.O.V.	Df	Plant Height (cm)	Tiller Number	Awn Length (cm)	Peduncle Length (cm)	Spike Length (cm)	Spike Weight (g)	Seed/Spike	No. of Spike Per m^2^	1000 Seed Weight	Grain Yield
Years	1	78.10 ns	42.83 **	3.50 **	20.23 ns	9.65 ns	0.06 ns	71.91 *	19,543.12 ns	81.64 **	2,676,365.6 ns
(Rep × Y)	6	150.20	1.43	5.70	44.11	175.39	0.08	289.90	155,331.04	30.33	3,459,259.0
Irrigation	2	1948.06 **	50.10 **	2.44 **	148.00 **	504.88 **	1.16 **	2808.48 **	596,113.58 **	408.36 **	53,654,858.4 **
(Y × I)	2	125.85 ns	2.17 ns	0.05 ns	164.79 **	0.10 ns	0.01 ns	24.57 ns	7041.89 ns	2.85 ns	5,372,535.7 **
(Ea)	12	227.15	16.67	2.63	103.20	75.56 ns	0.17 ns	528.86	524,607.66	163.30	19,089,250.7
Cultivars	1	91.64 ns	0.83 ns	25.34 **	5.31 ns	0.03 ns	0.05 ns	3.32 ns	87,912.94 **	65.81 **	1,986,721.5 ns
(I × V)	2	8050.84 **	0.11 ns	3.24 **	1097.59 **	111.78 **	0.01 ns	286.47 **	78,015.99 **	33.53 **	4,955,329.0 **
(Y ×C)	1	160.24 *	2.45 ns	1.40 *	13.99 ns	3.56 ns	0.00 ns	91.86 **	236.86 ns	3.28 ns	1,219,019.9 ns
(Y × I × C)	2	65.45 ns	0.72 ns	0.78 ns	89.16 ns	0.74 ns	0.00 ns	3.15 ns	26,999.93 ns	2.02 ns	12,621.5 ns
(Eb)	18	1734.36	1.46	4.42	176.03	28.52	0.14	88.50	237,633.88	77.44	4,268,545.1
Biofertilizers	7	42.23 ns	6.15 **	0.49 ns	41.27 ns	6.70 *	0.04 ns	41.06 **	71,092.60 ns	7.55 ns	2,129,087.8 **
(I × B)	14	67.44 **	2.43 ns	0.31 ns	27.27 ns	6.23 *	0.03 ns	20.30 **	235,828.83 **	9.51 **	1,763,713.7 **
(V × B)	7	21.71 ns	2.35 ns	0.19 ns	15.11 ns	7.67 *	0.05 *	16.92 ns	86,023.26 ns	6.31 ns	1,218,132.4 ns
(I × C × B)	14	17.94 ns	2.77 ns	0.40 ns	22.50 ns	3.50 ns	0.04 **	23.19 *	123,748.20 ns	7.39 *	1,196,413.4 ns
(Y × B)	7	0.46 ns	0.53 ns	0.21 ns	25.59 ns	0.55 ns	0.01 ns	6.80 ns	19,362.16 ns	0.60 ns	261,391.0 ns
(Y × I × B)	14	2.61 ns	0.28 ns	0.16 ns	29.10 ns	0.92 ns	0.00 ns	5.40 ns	51,946.39 ns	0.72 ns	456,271.3 ns
(Y × V × B)	7	1.46 ns	0.43 ns	0.49 ns	40.14 ns	0.28 ns	0.01 ns	8.10 ns	31,561.39 ns	0.78 ns	167,019.2 ns
(Y × I × C × B)	14	4.04 ns	0.31 ns	0.17 ns	30.67 ns	0.64 ns	0.01 ns	8.90 ns	73,792.74 ns	0.26 ns	371,956.2 ns
(Ec)	252	24.46	1.70	0.29	31.03	3.02	0.02	11.55	6509.04	3.73	708,173.2
(%) C.v.	-	4.65	21.73	11.18	14.31	11.30	12.06	12.46	13.44	4.68	19.33

** and *: significant in 1% and 5% level, respectively.

**Table 2:** Mean comparisons of dual interaction of year and irrigation on peduncle length in wheat.

Treatment (Year × Irrigation)	Peduncle Length (cm)	Grain Yield (kg/ha)
Dry farming × first year	39.14a	3687.66a
Irrigation once × first year	39.78b	4625.16a
Irrigation twice × first year	40.36ab	5003.75b
Dry farming × second year	37.04ab	3508.59b
Irrigation once × the second year	37.28b	4436.41a
Irrigation twice × second year	39.98ab	4856.88a

Means that have a common letter have not significantly different together (slicing, *p* < 0.05).

**Table 3:** Mean comparisons of the dual interactions of Years and cultivars on some traits.

Treatment (Year × Cultivars)	Plant Height (cm)	Awn Length (cm)	Seed/Spike
first year × Sardari cultivar	107.94a	4.96ab	27.28a
first year × Sirvan cultivar	105.94a	4.55b	28.12a
second year × Sardari cultivar	106.26a	5.24a	26.34a
second year × Sirvan cultivar	105.75a	4.62b	27.38a

Means that have a common letter have not significantly different together (slicing, *p* < 0.05).

**Table 4:** Mean comparisons of the dual interactions of irrigation and cultivars on some traits.

Treatment (Cultivars × Irrigation)	Plant Height (cm)	Awn Length (cm)	Peduncle Length (cm)	Spike Length (cm)	Seed/Spike	No. of Spike Per m^2^	1000-Seed Weight (g)	Grain Yield (kg/ha)
Sardari cultivar × Dry land farming	108.23ab	4.95ab	36.91b	13.13c	20.97e	527.38b	39.80c	3598.3b
Sirvan cultivar×Dryland farming	96.71b	4.80ab	36.78ab	14.00c	24.02de	518.66b	39.56c	3598.0b
Sardari cultivar × 1 Irrigation	114.05a	5.07ab	40.05ab	13.95c	25.89cd	631.09a	41.94abc	4425.2a
Sirvan cultivars × 1 Irrigation	98.81b	4.35b	36.78b	16.13b	28.97bc	659.09a	39.92bc	4643.1a
Sardari cultivar × 2 Irrigations	116.02a	5.32a	43.44a	16.85ab	31.55ab	637.25a	43.34a	4838.9a
Sirvan cultivar × 3 Irrigations	105.00ab	4.54b	40.20ab	18.20a	32.30a	641.33a	42.98ab	5015.0a

Means that have a common letter have not significantly different together (slicing, *p* < 0.05). Different letters indicate significant differences in the values

**Table 5:** Mean comparisons of triple interactions of cultivars × irrigation ×Biofertilizers on some traits (Means that have a common letter have not significantly different (*p* < 0.05).

Treatment (Cultivars × Irrigation × Biofertilizers)	Spike Weight (g)	Seed/Spike	1000-Seed Weight (g)
Sardari × dry land farming × control	0.96 o	19.13 s	38.38 stu
Sardari× dryland farming × mycorrhiza	1.02 lmno	19.75 rs	39.50 opqrstu
Sardari × dryland farming × seaweed extract	1.06 ijklmno	21.00 qrs	39.50 opqrstu
Sardari × dry land farming × nitrozist and phosphozist	1.02 lmno	21.00 qrs	39.75 mnopqrst
Sardari × dryland farming × mycorrhiza + seaweed extract	1.04 klmno	21.13 qrs	40.00 lmnopqrst
Sardari × dryland farming × mycorrhiza + nitrozist and phosphozist	1.08 ghijklmno	21.75 opqrs	40.38 jklmnopqr
Sardari × dryland farming × nitrozist and phosphozist + seaweed extract	1.10 fghijklmn	21.38 pqrs	40.63 lmnopqr
Sardari × dryland farming × mycorrhiza + seaweed extract + nitrozist and phosphozist	1.12 efghijklmn	22.63 nopqr	40.88 hijklmnop
Sirvan × dryland farming × control	1.01 no	21.38 pqrs	37.75 u
Sirvan × dryland farming × mycorrhiza	1.02 lmno	21.63 opqrs	38.88 rstu
Sirvan × dryland farming × seaweed extract	1.02 lmno	23.38 mnopq	39.13 qrstu
Sirvan × dryland farming × nitrous and phosphate	1.02 mno	23.63 lmnopq	39.13 qrstu
Sirvan × dryland farming × mycorrhiza + seaweed extract	1.03 lmno	23.75 lmnopq	39.63 nopqrst
Sirvan × dryland farming × mycorrhiza + nitrozist and phosphozist	1.06 jklmno	24.13 klmnopq	39.75 mnopqrst
Sirvan × dryland farming × seaweed extract + nitrozist and phosphozist	1.11 fghijklmn	26.38 ijklm	39.63 nopqrstu
Sirvan × dryland farming × mycorrhiza + seaweed extract + nitrozist and phosphozist	1.13 defghijklmn	27.88 fghij	40.25 jklmnopqrs
Sardari × irrigation once × control	1.01 mno	24.63 jklmnop	40.88 hijklmnopq
Sardari × irrigation once × mycorrhiza	1.06 jklmno	24.75 jklmno	41.38 fghijklmno
Sardari × irrigation once × seaweed extract	1.09 ghijklmno	25.75 jklmn	41.63 efghijklm
Sardari × irrigation once × nitrozist and phosphozist	1.14 defghijklmn	25.88 jklmn	41.25 fghijklmno
Sardari × irrigation once × mycorrhiza + seaweed extract	1.14 defghijklmn	26.00 jklm	42.00 cdefghijk
Sardari × irrigation once × mycorrhiza + nitrozist and phosphozist	1.14 defghijklm	26.25 jklm	42.38 bcdefghi
Sardari × irrigation once × nitrozist and phosphozist + seaweed extract	1.15 cdefghijkl	26.50 ijklm	43.00 abcdefg
Sardari × irrigation once × mycorrhiza + seaweed extract + nitrozist and phosphozist	1.15 cdefghijkl	27.38 ghijk	43.00 abcdefg
Sirvan × irrigation once × control	1.07 hijklmno	25.38 jklmn	38.25 tu
Sirvan × irrigation once × mycorrhiza	1.09 ghijklmno	26.50 ijklm	38.75 rstu
Sirvan × irrigation once × seaweed extract	1.11 efghijklmn	26.75 hijkl	39.13 qrstu
Sirvan × irrigation once × nitrous and phosphate	1.17 cdefghijk	29.88 defgh	39.25 pqrstu
Sirvan × irrigation once × mycorrhiza + seaweed extract	1.18 cdefghij	30.00 defgh	40.13 lmnopqrst
Sirvan × irrigation once × mycorrhiza + nitrozist and phosphozist	1.19 cdefghij	30.50 bcdefg	40.88 ijklmnopq
Sirvan × irrigation once × seaweed extract + nitrozist and phosphozist	1.20 cdefghi	30.88 bcdef	41.50 fghijklmn
Sirvan × irrigation once × mycorrhiza + seaweed extract + nitrozist and phosphozist	1.20 cdefgh	30.75 bcdef	41.50 fghijklmn
Sardari × irrigation twice × control	1.07 hijklmno	29.63 efgh	41.13 ghijklmnop
Sardari × irrigation twice × mycorrhiza	1.19 defghij	30.88 bcdef	41.88 efghijkl
Sardari × irrigation twice × seaweed extract	1.120 fghijklmn	31.00 bcdef	42.13 cdefghij
Sardari× irrigation twice × nitrozist and phosphozist	1.25 cde	32.50 abcde	42.75 abcdefgh
Sardari × irrigation twice × mycorrhiza + seaweed extract	1.24 cdef	32.75 abcde	43.50 abcde
Sardari × irrigation twice × mycorrhiza + nitrozist and phosphozist	1.27 cd	33.50 abc	43.75 abcd
Sardari × irrigation twice × nitrozist and phosphozist + seaweed extract	1.28 bc	33.75 a	44.13 ab
Sardari × irrigation twice × mycorrhiza + seaweed extract + nitrozist and phosphozist	1.44 a	34.75 a	44.63 a
Sirvan × irrigation twice × control	1.13 defghijklmn	29.88 defgh	41.88 defghijkl
Sirvan × irrigation twice × mycorrhiza	1.19 cdefghij	30.13 defg	43.13 abcdef
Sirvan × irrigation twice × seaweed extract	1.22 cdefg	30.25 cdefg	43.13 abcdef
Sirvan × irrigation twice × nitrous and phosphate	1.24 cdef	30.88 bcdef	43.13 abcdef
Sirvan × irrigation twice × mycorrhiza + seaweed extract	1.25 cde	32.25 abcde	43.63 abcd
Sirvan × irrigation twice × mycorrhiza + nitrozist and phosphozist	1.28 bc	31.13 bcdef	43.75 abcd
Sirvan × irrigation twice × seaweed extract + nitrozist and phosphozist	1.29 bc	33.00 abcd	43.88 abc
Sirvan × irrigation twice × mycorrhiza + seaweed extract + nitrozist and phosphozist	1.42 ab	34.88 a	44.25 ab

Means in each column followed by similar letter(s) are not significantly different at 1% probability level by Lsd test.

## Data Availability

All the data is present in the manuscript and it’s supplementary files.

## Supplementary Materials

The following are available online at https://www.mdpi.com/article/10.3390/plants10040811/s1, Table S1: Physicochemical properties of the soil before planting in two years of experiment and Table S2. The correlation coefficient of studied traits in wheat.
