# Supplementary material for: Effects of the Combinations of Rhizobacteria, Mycorrhizae, and Seaweed, and Supplementary Irrigation on Growth and Yield in Wheat Cultivars
Source: Plants (Basel). 2021 Apr 20;10(4):811. doi: 10.3390/plants10040811 (PMC8074330; doi:10.3390/plants10040811)
Supplement: Supplementary file 1 [file plants-10-00811-s001.zip › plants-1162744-supplementary.pdf]

Article

Effects of the Combinations of Rhizobacteria, Mycorrhizae, and Seaweed, and Supplementary Irrigation on Growth and Yield in Wheat Cultivars

Z. Najafi Vafa [1,*], Y Sohrabi [1], R. Z. Sayyed [2], Ni Luh Suriani [3] and Rahul Datta [4,*]

Table 1. Physicochemical properties of the soil before planting in two years of experiment.

Soil Characteristics	2017–2018	2018–2019
Depth of Soil (cm)	0–30	0–30
Soil Texture	Loam	Clay
Electrical Conductivity (dsm^{-1})	362	0.505
pH	7.17	7.18
Available K (mg/Kg)	214.18	250.80
Available P (mg/Kg)	14.87	18.2
Total Organic Carbon (%)	4.91	5.33
Total Nitrogen (%)	0.105	0.137

Table 2. The correlation coefficient of studied traits in wheat.

	Plant Height	Tiller Number	Spike Length	Spike Weight	Seed/Spike	No. of Spike/m²	1000-Seed wt	Grain Yield
Plant Height	1							
Tiller Number	0.47 **	1						
Spike Length	0.03 ns	−0.11 ns	1					
Spike Weight	0.02 ns	0.03 ns	0.62 **	1				
(Seed/Spike)	0.43 **	0.47 **	−0.15 *	−0.01 ns	1			
No. of Spike/m²	0.59**	0.28 **	−0.17 *	−0.20 **	0.39 **	1		
1000 Seed Weight	0.51 **	0.44 **	0.04 ns	0.09 ns	0.68 **	0.43 **	1	
Grain Yield	0.14 ns	−0.05 ns	−0.31 **	−0.29 **	0.07 ns	0.52 **	−0.04 ns	1

** and *: significant in 1% and 5% level, respectively.
